# Collimated and non-collimated proton minibeam irradiation using SIRMIO: a simulation study

**DOI:** 10.2340/1651-226X.2025.44031

**Published:** 2025-11-02

**Authors:** Fardous Reaz, Ze Huang, Marco Pinto, Jonathan Bortfeldt, Niels Bassler, Katia Parodi

**Affiliations:** a Department of Clinical Medicine, Aarhus University, Aarhus, Denmark; b Danish Centre for Particle Therapy, Aarhus University Hospital, Aarhus, Denmark; c Department of Medical Physics, Ludwig-Maximilians Universität München (LMU Munich), Munich, Germany

**Keywords:** pMBRT, SIRMIO, PVDR

## Abstract

**Background and purpose:**

Successful clinical integration of pMBRT requires comprehensive investigations of the relationship between various pMBRT parameters and their associated biological effects. Such investigations are critically dependent on small animal models. Therefore, a state-of-the-art small animal irradiation platform like SIRMIO (Small Animal Proton Irradiator for Research in Molecular Image-guided Radiation-Oncology), capable of delivering precisely controlled spatially fractionated doses, is highly desirable for advancing preclinical pMBRT research.

**Material and methods:**

This in silico study evaluates the SIRMIO beamline’s capability to deliver beams essential for pMBRT experiments. We used Geant4-based Monte Carlo simulations to investigate two configurations: one without a collimator, and one using a 30 mm thick brass multislit collimator (MSC). For both configurations, we examined center-to-center (CTC) of 3, 4, and 5 mm, with a constant 1 mm slit width when MSC is used.

**Results:**

The SIRMIO beamline can effectively generate spatially fractionated dose profiles with varying CTC. Without a collimator, sufficient dose contrast for pMBRT can be achieved with CTC of 4 mm and above, as evidenced by peak-to-valley dose ratios (PVDR) of 3.44 and 6.57 for 4 and 5 mm CTC, respectively. MSC further enhances dose contrast, achieving PVDR of 11.3, 20.7, and 28.7 for 3, 4, and 5 mm CTC, respectively. Furthermore, we explored interlacing beams as a means of achieving a uniform target dose while preserving dose contrast in normal tissue, demonstrating the potential of this approach using the SIRMIO beamline.

**Interpretation:**

The SIRMIO platform can be a viable option for pMBRT experiments.

## Introduction

External beam radiotherapy (EBRT) is an essential element of modern cancer treatment, widely employed for both curative and palliative purposes [1, 2]. However, despite its effectiveness, EBRT inevitably exposes healthy tissues to radiation, leading to the risk of side effects. Minimizing normal tissue toxicity while achieving optimal tumor control remains a critical goal in radiotherapy. Emerging approaches like FLASH and spatially fractionated radiotherapy (SFRT) have shown promise in reducing radiation-induced damage to healthy tissues while maintaining or enhancing tumor control [3–9].

SFRT, which delivers highly heterogeneous doses, offers a unique balance between minimizing normal tissue toxicity and improving tumor response. It is categorized into GRID radiotherapy, LATTICE radiotherapy, minibeam radiotherapy (MBRT), and microbeam radiotherapy (MRT) based on beam size, spacing, and dose distribution pattern [10]. Among these, MBRT utilizes millimeter-spaced beam arrays to create periodic patterns of high-dose peaks and low-dose valleys. This spatial dose modulation significantly reduces damage to healthy tissues while preserving tumor control [11]. Although the exact biological mechanisms underlying SFRT remain incompletely understood, they are hypothesized to involve the dose-volume effect, radiation-induced bystander effects, microvascular changes, and immunomodulation [12–16].

Proton therapy has emerged over the last two decades as a promising modality to complement conventional photon-based radiotherapy through enhanced dose conformity and sparing healthy tissues. Its superior depth-dose profile enables a more localized dose deposition, reducing the integral dose to healthy tissues by a factor of 2–3 compared to photon therapy [17]. Despite its advantages, proton therapy faces limitations in specific clinical contexts. For instance, dose escalation in radioresistant tumors is often limited by normal tissue toxicity [18, 19]. Similarly, re-irradiation of recurrent tumors is restricted due to exceeding the tolerance threshold of previously irradiated tissues [20–22]. These challenges are particularly pronounced in pediatric cases, where minimizing radiation exposure is essential to preserve long-term quality of life [23–25]. Addressing these limitations requires advances that can extend the therapeutic window of proton therapy.

Proton minibeam radiotherapy (pMBRT) is a promising innovation aimed at overcoming these constraints. By combining the spatial dose modulation principles of MBRT with the dosimetric benefits of proton therapy, pMBRT offers the potential to reduce normal tissue toxicity and achieve superior tumor control [26]. Preliminary studies have demonstrated its advantages over conventional proton therapy, though its clinical efficacy has yet to be validated [27–31]. Several parameters specific to spatially fractionated techniques, such as the average dose, peak dose, valley dose, and peak-to-valley dose ratio (PVDR), are linked to biological outcomes of SFRT [32, 33]. However, further evaluation is necessary to precisely understand the role of these parameters and the biological mechanisms underlying pMBRT. Since most pMBRT research relies on small animal models, a dedicated platform is highly desirable to advance the reproducibility and accuracy of these radiobiological experiments.

To explore the potential of pMBRT and its biological mechanism, the SIRMIO (Small Animal Proton Irradiator for Research in Molecular Image-guided Radiation-Oncology) image-guided proton irradiation platform, developed over the last years at Ludwig-Maximilians Universität München [34] and extensively tested at the Danish Centre for Particle Therapy (DCPT), Aarhus, Denmark, offers a state-of-the-art system for precision small animal radiation research. The SIRMIO beamline is specifically designed for precise and adaptable proton delivery, integrating advanced features such as magnetic focusing for (sub)millimeter beam spot sizes (sigma in air at isocenter), energy modulation, and innovative imaging techniques. The platform’s current on-board imaging capabilities include proton radiography and real-time range verification using PET imaging, ensuring precise tumor targeting and dose delivery. In addition, the system incorporates a custom mouse holder that ensures stability and physiological support during experiments, improving the quality of preclinical research. As a portable and adaptable system, SIRMIO can bridge the gap between preclinical research and clinical application, making it a powerful tool to investigate the potential of pMBRT.

In this work, in silico, we performed an evaluation of the SIRMIO beamline for pMBRT applications using two complementary approaches: non-collimated beam optics modifications and the application of a slit collimator. The non-collimated approach depends on the magnetic focusing to create precise beam profiles, while the slit collimator generates a spatially modulated dose distribution through a static mechanical design. Using Monte Carlo simulations, this study performs an assessment of the SIRMIO beamline’s capabilities for pMBRT studies. It proposes essential modifications that might be implemented for future experimental use in pMBRT.

## Material and methods

The dose distribution in pMBRT is highly sensitive to beam parameters, especially when a collimator is employed [35]. The portable SIRMIO platform is designed for integration at various proton therapy facilities [36]. Its active beamline segment comprises a boron carbide degrader for energy modulation, a graphite collimator system for beam shaping, and a triplet of permanent quadrupole magnets for beam parameter tuning [37]. An incoming beam of 70 MeV to 100 MeV is passively and actively shaped to achieve the desired energy and beam parameters for focusing at a predefined point. To reduce complexity and computation time, we did not simulate the entire beamline; instead, we started from the phase-space of the experimentally validated beam model [38].

### Simulation objective

**Figure 1:**
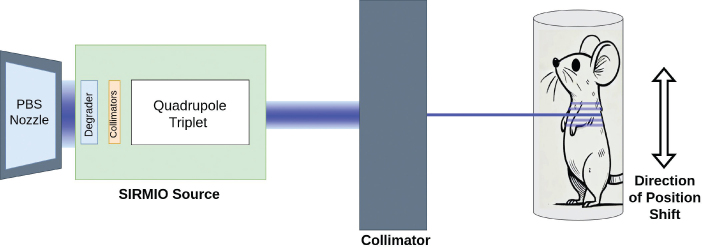
Schematic representation of the pMBRT experimental setup using the SIRMIO (Small Animal Proton Irradiator for Research in Molecular Image-guided Radiation-Oncology) beamline. A dedicated low-energy proton beam, produced by the SIRMIO platform and additionally shaped by a collimator, irradiates a target (mouse). The distance between the nozzle and the SIRMIO source ranges from 30 cm to 50 cm, while the pMBRT collimator is positioned approximately 1 cm upstream of the target. Precise vertical translation of the target holder on stages enables the delivery of multiple, spatially separated minibeams, creating a peak-valley dose distribution pattern.

The goal of this study is to evaluate, through simulations, the capability of the SIRMIO beamline to generate pMBRT dose distributions. We aim to produce spatially fractionated dose profiles characterized by alternating high-dose peaks and low-dose valleys. As shown in Figure 1, the SIRMIO setup directs a proton beam toward a target mounted on translational–rotational stages. It employs a motorized system that enables precise movement and rotation of the stage, with step sizes of 10 µm and 0.1°, respectively. By adjusting the vertical position of the stage, the characteristic peak–valley dose pattern can be generated, where the CTC between peaks is determined by the magnitude of the vertical shift. We perform simulations for both a collimator configuration and a non-collimated configuration. In each case, we quantify the achievable PVDR and overall dose distribution characteristics across a range of CTC.

### Beam generation and simulation setup

We used a pre-generated, validated phase-space file, which defines each particle’s spatial, momentum, and energy distributions at a fixed position, as a virtual source [38]. Protons with KE more than 0.1 MeV and located within a 200 mm radius were transported. We sampled particles uniformly and applied Y-direction shifts (based on beamlet index and chosen CTC) to recreate the parallel beamlet geometry used in pMBRT, equivalent to moving the target holder. This study used Geant4 version 11.0.3 [39] for all simulations. The physics processes were modeled using the ‘QGSP_BIC_HP_EMY’ physics list, which combines the Quark–Gluon String Precompound (QGSP) model, the Binary Cascade (BIC) model, and the High Precision (HP) neutron package for low-energy neutron transport, and was found a suitable choice for simulating proton beams of less than 200 MeV. In addition, our simulation framework activates G4DecayPhysics, G4StoppingPhysics, and G4IonPhysics. The EMY option provides an improved electromagnetic physics configuration suitable for medical and dosimetric applications. Overall our setting enables us to achieve a reasonable balance between accuracy and computational efficiency in hadron therapy simulations [40]. A production cut of 0.05 mm was employed. Each simulation involved the transport of 10^8^ primary particles to minimize statistical fluctuations, ensuring that the uncertainty in the valley dose remained below 1%.

### Scoring

We recorded various quantities, including dose distributions in materials (air and water), energy spectra, and phase-space information. The simulations did not include any specific target or mouse geometry. Instead, the dose distribution was scored in air at the position corresponding to the mouse location during irradiation. This approach was chosen because the focus of the study was to characterize the spatial dose modulation of the beam at the entrance of the mouse body, rather than the dose deposition within individual organs. Therefore, quantification in air reliably represents the entrance dose pattern. Dose distributions in water were also evaluated to estimate how the beam modulates in a tissue-equivalent medium. One-dimensional dose histograms were created with 1,000 bins, spanning a range of -50 mm to +50 mm, resulting in a bin width of 0.1 mm along the direction of the dose fractionation. Two-dimensional dose distributions in the XY plane utilized 1,000 bins in both the X and Y dimensions, also covering a range of -50 to +50 mm. High-resolution scoring for these 2D maps used a voxel size of 0.1 × 0.1 mm, with a 1 mm extension in the third dimension. To characterize the beam’s phase-space, we generated two 2D histograms: one for the X-X’ plane (600 bins for position and divergence, covering -30 to +30 mm and -0.3 to +0.3 mrad, respectively) and one for the Y-Y’ plane (300 bins for position [-15 to +15 mm], 200 bins for divergence [-0.1 to +0.1 mrad]). Finally, additional histograms were generated to record depth-dose profiles (IDD) and 2D dose maps in the YZ plane with a larger bin size along depth.

### Collimator

The simulations incorporated a brass multislit collimator (MSC) design with a thickness of 30 mm, ensuring complete proton stopping for the energies considered. The collimator with various CTCs was simulated, maintaining a constant aperture of 1.0 mm unless otherwise stated. This MSC could be replaced by a monoslit collimator in a physical experiment.

## Results

**Figure 2:**
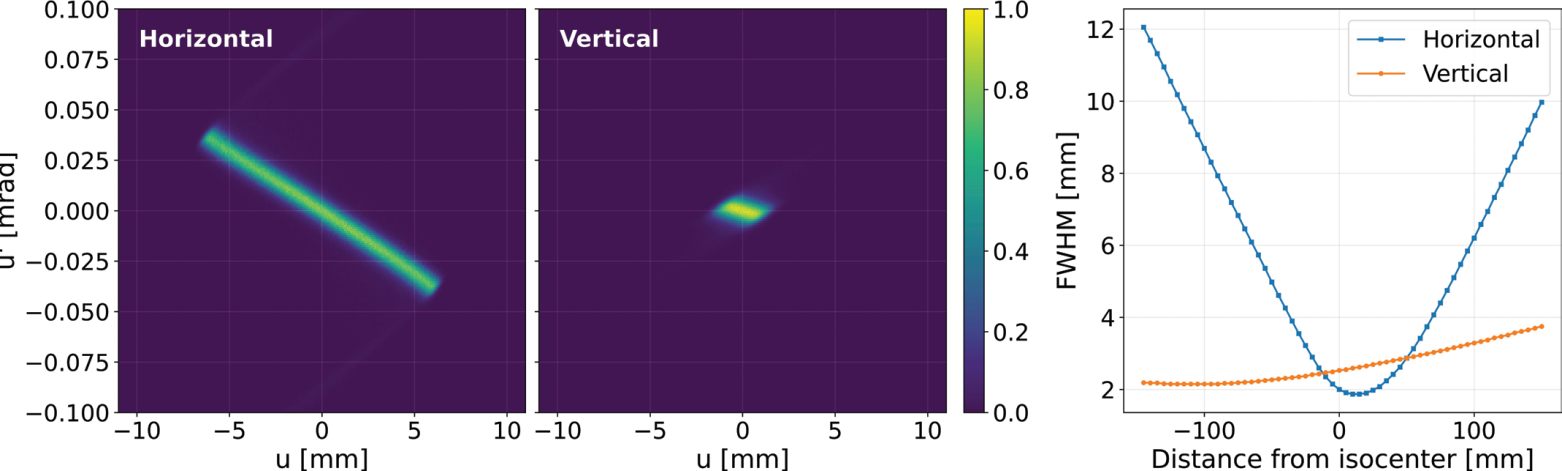
Initial horizontal (left) and vertical (center) phase-space distributions of the 50 MeV proton beam at the virtual source position. Evolution of the beam size (FWHM) in the horizontal and vertical planes as a function of distance from the isocenter (Right).

In simulations, a virtual source was placed 148.08 mm upstream of the isocenter of the SIRMIO platform after the SIRMIO source. The initial horizontal and vertical components of phase-space of the 50 MeV proton beam are shown in Figure 2 (left and center). These two phase-spaces exhibit distinct characteristics: the vertical phase-space distribution is notably narrower in both spatial and angular extent compared to the horizontal distribution. This observation indicates a significantly larger emittance in the horizontal plane according to the SIRMIO beamline design with magnetic focusing/defocusing elements [37].

As the beam propagates in air, its phase-space evolves as determined by the magnet quadrupole triplet of SIRMIO source, which focuses the beam towards the isocenter. The variation in beam size (full width at half maximum, FWHM) in both horizontal and vertical directions as a function of position is depicted in Figure 2 (right). A significant difference is observed between the two planes; the horizontal beam size undergoes a rapid reduction from 12 mm at -148.08 mm to a minimum of 2.0 mm just after the isocenter, followed by a pronounced expansion. In contrast, the vertical beam size increases gradually from its initial value of 2.0 mm, with a growth constrained to less than 2.5 mm up to the isocenter.

**Figure 3:**
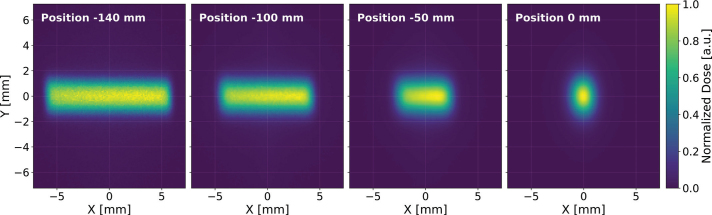
Dose distributions in air by the 50 MeV proton beam at -140 mm, -100 mm, -50 mm, and (d) 0 mm from the isocenter of SIRMIO (Small Animal Proton Irradiator for Research in Molecular Image-guided Radiation-Oncology) platform. The relative dose normalized to the maximum value. The beam shape transitions from a rectangular geometry upstream to a circular profile at the isocenter.

**Figure 4:**
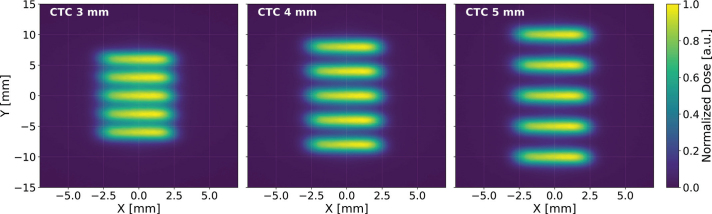
Two-dimensional dose distribution at -50 mm upstream of the SIRMIO (Small Animal Proton Irradiator for Research in Molecular Image-guided Radiation-Oncology) isocenter for different center-to-center distnaces (CTCs): (left) 3 mm, (center) 4 mm, and (right) 5 mm. The dose patterns are generated by vertically shifting the target holder in the SIRMIO platform. The color scale represents the normalized dose.

Figure 3 presents dose distributions illustrating the beam spot at four distinct positions along the SIRMIO beamline in air. At the isocenter (0 mm), the beam profile is approximately symmetric, exhibiting similar spatial extent in the two orthogonal directions. However, at upstream locations, the beam shape evolves towards a rectangular geometry, most prominently between -140 and -100 mm. This elongation creates a narrow dose distribution peak, characteristic of the profiles employed in pMBRT using MSCs. This inherent beam-shaping capability suggests the SIRMIO beamline is well-suited for delivering non-collimated spatially fractionated dose profiles with minimal or no additional beam modification. The target animal is placed on a movable holder in the SIRMIO platform, enabling precise vertical translation and rotation. A key feature of pMBRT, the periodic peak-valley dose distribution, can be generated by incrementally shifting the target vertically between each dose delivery. This precise vertical shift directly corresponds to the desired CTC, a critical parameter optimized according to the experimental design. Figure 4 shows 2D dose profiles acquired in air at a position 50 mm upstream of the isocenter. Profiles are shown for CTC of 3, 4, and 5 mm, with dose values normalized to the maximum. The characteristic peaks and valleys clearly demonstrate the system’s capability to precisely control the spatial fractionation of the dose by simple adjustments to the platform’s vertical position. While a 4 mm and above CTC yields sufficient dose contrast to observe the tissue-sparing effect of the spatially fractionated dose distribution, further contrast enhancement can be achieved, even with a 3 mm CTC, by incorporating a collimator into the beamline. This collimator, either a monoslit or multislit design, remains stationary with its aperture positioned at a specific vertical location, preferably at the isocenter plan. The animal holder can move in independent steps synchronized with the delivery, allowing the beam to scan across the target, while the collimator effectively trims the lateral Gaussian tails of the beam. This trimming action reduces the dose in the valleys between minibeams, thereby increasing the lateral dose gradient and improving the PVDR.

**Figure 5:**
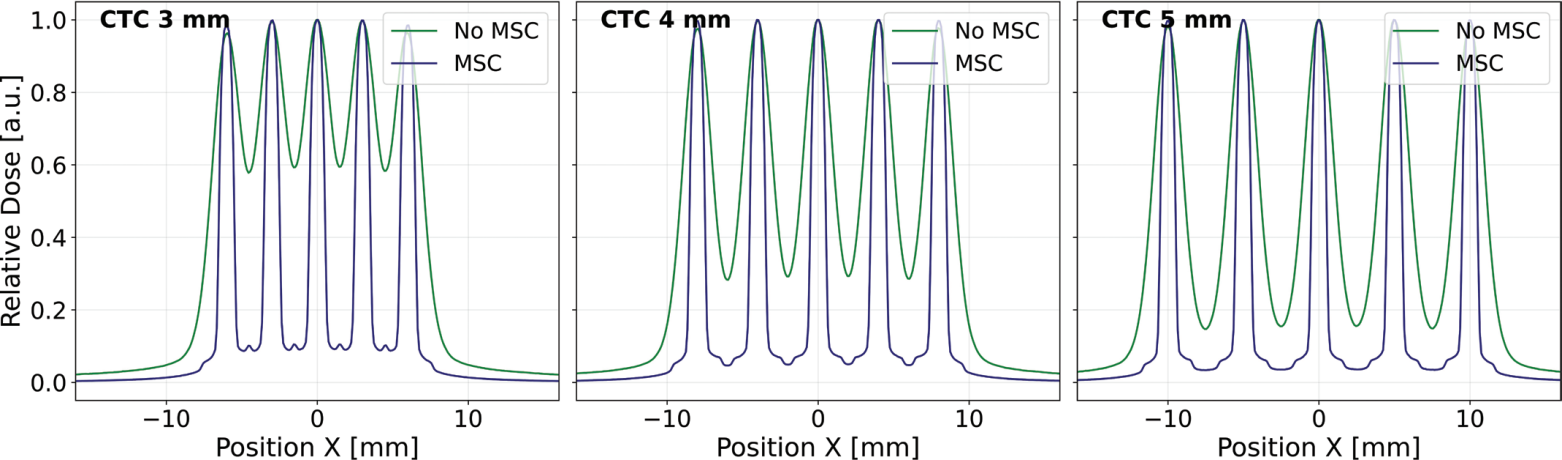
One-dimensional dose distributions in air at -50 mm upstream of the SIRMIO (Small Animal Proton Irradiator for Research in Molecular Image-guided Radiation-Oncology) isocenter for center-to-center distance (CTC) of 3, 4, and 5 mm. The collimator enhances the peak-to-valley dose ratio (PVDR) for all CTCs.

Figure 5 presents a comparison of 1D dose profiles obtained with and without a collimator for CTC of 3, 4, and 5 mm at 50 mm upstream of the isocenter. As evident in the figure, the introduction of the collimator in the beamline leads to a substantial increase in PVDR for all CTC assessed here. This improvement in dose contrast underscores the collimator’s effectiveness in further shaping the dose distribution and maximizing the contrast for pMBRT irradiation.

Table 1 presents a comparison of the average peak dose, average valley dose, and PVDR for collimated and non-collimated configurations for different CTC values. Without MSC, the PVDR at 3 mm CTC is 1.68, indicating a limited dose contrast. Increasing the CTC to 4 mm and 5 mm improves the PVDR to 3.44 and 6.57, respectively. However, the introduction of the MSC significantly elevates the PVDR for all CTC values. With MSC, the PVDR at 3 mm CTC increases to 11.3. Even higher PVDR values are achieved for 4 mm and 5 mm CTC with the MSC, reaching 20.7 and 28.7, respectively.

**Table 1:** Comparison of average peak dose, average valley dose, and PVDR for MSC and no MSC data.

CTC (mm)	Group	Avg. peak dose	Avg. valley dose	PVDR
3	MSC	1.0	0.09	11.3
3	No MSC	0.99	0.59	1.7
4	MSC	1.0	0.05	20.7
4	No MSC	0.99	0.29	3.4
5	MSC	1.0	0.04	28.7
5	No MSC	0.99	0.15	6.6

**Figure 6:**
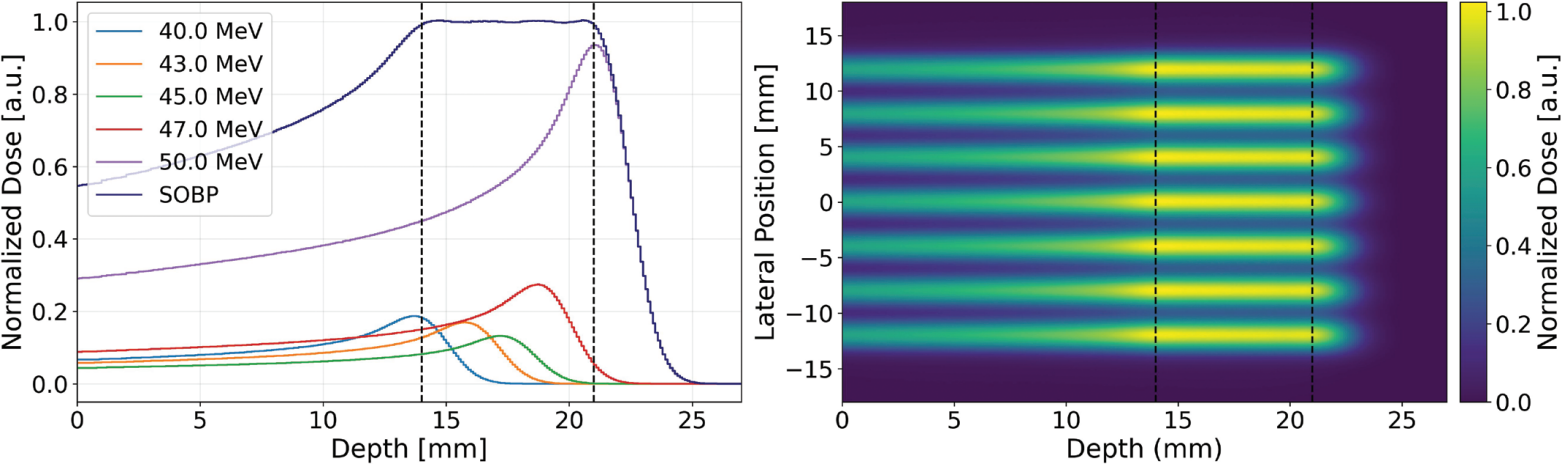
Depth-dose profiles for individual beam energies (40.0, 43.0, 45.0, 47.0, and 50.0 MeV) and the resulting Spread-Out Bragg Peak (SOBP) (left). The shaded region indicates the longitudinally uniform dose region. Two-dimensional dose distribution of the SOBP extending from a depth of 14 to 21 mm. The color scale represents the normalized dose (right).

A monoenergetic beam, with the target placed within the initial plateau of the depth-dose curve, may be sufficient to study normal tissue sparing. However, for tumor control studies, a longitudinally uniform dose across the target volume is often desirable. We therefore created a Spread-Out Bragg Peak (SOBP) by combining five different beam energies. The optimized weight for each beam energy, crucial for achieving the desired uniformity, is given in Table 2. Figure 6 (left) illustrates the resultant SOBP, which provides a 7 mm uniform dose region (extending from 14 to 21 mm depth in water). The figure also shows the individual Bragg peaks contributing to the final SOBP.

**Table 2:** Beam energies and corresponding weights used to generate the SOBP.

Beam energy [MeV]	40.0	43.0	45.0	47.0	50.0
Weight	0.19	0.17	0.13	0.27	0.94

**Figure 7:**
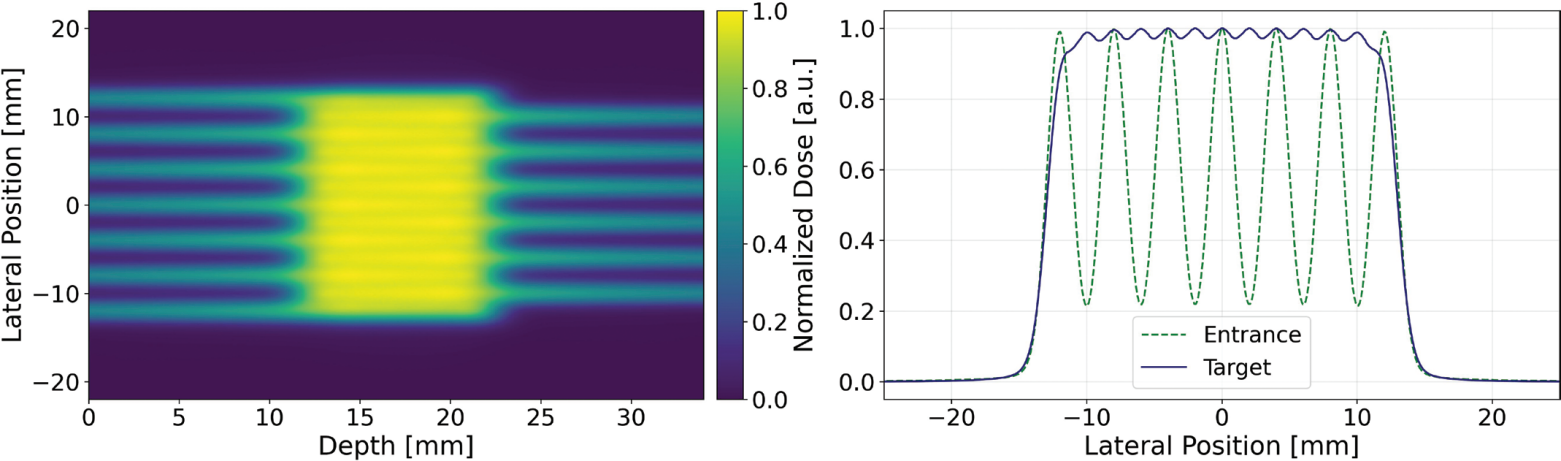
Two dimensional dose distribution for an interlaced beam configuration with a center-to-center distance (CTC) of 4 mm (Left). Dose profile at the entrance and within the SOBP region (Right).

Given that the maximum beam energy of SIRMIO is 50 MeV, the proton range in water is limited to approximately 22 mm. Consequently, lateral broadening of proton beams within the target is insufficient to produce a laterally homogeneous dose using an array of unidirectional parallel beams, as illustrated in Figure 6 (right). The 2D dose distribution at any depth exhibits pronounced peaks and valleys, characteristic of the individual beam contributions. This spatially heterogeneous dose profile, while not ideal for achieving a uniform field, presents a potential advantage for fractionated target irradiation studies. Optimal treatment outcomes might require achieving both a uniform dose distribution within the target volume and a high dose contrast in the surrounding normal tissue. This approach could maintain tumor control efficacy the same as conventional modality while minimizing damage to healthy tissues. A significant challenge arises when using a single array of unidirectional beams, as the CTC necessary for adequate dose contrast at the entrance region leads to a non-uniform dose distribution within the target, as shown in Figure 6 (right). To overcome this limitation, the feasibility of delivering a homogeneous target dose using the SIRMIO beamline was investigated through the implementation of interlaced beams, comprising two oppositely propagating proton beam arrays. Figure 7 (left) shows a uniform dose achieved in the target through the implementation of two oppositely propagating arrays of non-collimated beams, employing a CTC of 4 mm. The underlying principle of this interlacing approach is to create a uniform dose distribution within the target volume using the superposition of peaks and valleys from the individual beams. The presence of a collimator, however, would necessitate a reduction in the CTC due to the significantly steeper lateral dose gradients it produces. The dose profile in the entrance region, as illustrated in Figure 7 (right), reveals the distinct peaks of the individual beams, producing a PVDR greater than 5. While this PVDR could be further increased, the primary objective of target dose homogeneity is achieved as the beams penetrate deeper. The overlap of peaks and valleys of two opposite beam arrays in the target region results in a uniform dose distribution. This observation underscores the efficacy of the interlacing technique in achieving a uniform target dose within the SIRMIO platform.

## Discussion and conclusion

Advancements in proton therapy are continually sought to improve treatment outcomes, and pMBRT represents a potentially transformative approach. Preclinical studies have shown pMBRT’s ability to reduce normal tissue toxicity while maintaining, or potentially improving, tumor control [27, 41]. However, further research is needed to understand the underlying biological mechanisms and optimize treatment parameters for clinical translation. This requires precise and reproducible experiments that often involve small animal irradiation. Small animal models are indispensable for understanding the complex interplay between pMBRT parameters and biological responses in both normal tissue sparing and tumor control [27–44]. However, the inherent challenges of precise placement, motion control, measurement, and imaging in small animals, particularly at the millimeter scale, require advanced irradiation platforms. The SIRMIO system directly addresses this need, providing a state-of-the-art platform with advanced capabilities, including precise beam control, magnetic focusing, energy modulation, and innovative imaging techniques [34, 37].

This study employed a dual methodology, utilizing both non-collimated beam and an MSC, to comprehensively assess the SIRMIO beamline’s capabilities for conducting pMBRT experiments, with a specific focus on its capability to generate spatially fractionated dose profiles with sufficient contrast. The results demonstrate that even in the absence of a collimator, the SIRMIO beamline achieves a PVDR of 6.6 at CTC of 5 mm. This level of dose contrast is comparable to that observed to be effective in SFRT modalities, commonly for GRID and LATTICE studies and often for pMBRT [10, 32, 45]. Furthermore, the incorporation of a collimator for smaller CTC significantly enhances PVDR, exceeding values of 10 and allowing finer spatial fractionation. This observed level of dose contrast is consistent with PVDR commonly used in pMBRT studies [45]. These findings confirm the suitability of the SIRMIO beamline for conducting critical investigations necessary to advance pMBRT.

Although non-collimated pMBRT typically exhibits lower dose contrast compared to MSC-based approaches, it presents several distinct advantages. A primary benefit is the ability to dynamically adjust the CTC without the need for multiple, physically distinct collimators, thus facilitating parameter studies [35, 46]. Furthermore, non-collimated configurations circumvent the stringent alignment requirements of MSCs, where minor misalignments can induce significant dose profile distortions [35], and avoid the issue of collimator activation and consequent secondary neutron and photon generation [47, 48]. Utilizing all incident protons in non-collimated beams can enhance system efficiency, which is particularly advantageous for achieving uniform target dose distributions and reducing the irradiation time, considering lower dose rates of small animal irradiation platforms. Future optimization of the SIRMIO system holds the potential to achieve higher dose contrast without the use of collimators, opening new possibilities for exploring non-collimated focused beam pMBRT, a technique that has shown promise compared to MSC approaches [49].

The study also identified a limitation associated with the maximum beam energy and available energy range of the current SIRMIO system, which is tailored to mouse irradiation. While the 7 mm SOBP achieved with the five-beam configuration may be sufficient for many small animal studies, there is a potential to extend this range by adding more energy layers. Lower beam energy reduces penetration depth and limits lateral broadening, making it harder to achieve uniform coverage with a single field. However, this limitation may also be viewed as an opportunity, as the resulting spatially fractionated dose profile could be beneficial to investigate the impact of dose heterogeneity on tumor response and its association with the dose distribution patterns. The non-collimated approach offers flexibility and simplicity, while the MSC approach ensures high dose contrast and precise beam shaping, depending on proper alignment. The choice between these methods depends on the specific requirements of the pMBRT study.

Although the simulations focused on dose distributions at a position 50 mm upstream of the isocenter, it is important to note that further upstream locations could yield increased lateral beam dimensions. The SIRMIO beamline’s quadrupole triplet [37] provides a mechanism for precise beam profile optimization. Therefore, it may be feasible to generate a horizontally larger beam spot size at the isocenter while maintaining a constrained vertical dimension (corresponding to the direction of fractionation). This could offer higher PVDRs at even smaller CTC, a potential avenue for future investigation.

To address the challenge of achieving a uniform target dose in larger volumes or in clinical scenarios that require homogeneous irradiation, the technique of interlacing beams, previously explored in pMBRT research [50], was investigated. Our simulation results demonstrate the efficacy of this approach in producing a uniform dose distribution within the target volume while maintaining dose contrast in the surrounding normal tissues. Here, the SIRMIO beamline offers a novel opportunity to experimentally validate the interlacing concept, as most prior work has been simulation-based.

The findings of this study open intriguing perspectives for preclinical pMBRT research. The demonstrated ability of the SIRMIO system to generate spatially fractionated dose profiles of varying spatial resolution and dose contrast, especially the flexibility to adjust CTC without needing to change collimators, opens new avenues for investigating the underlying biological mechanisms and optimizing treatment parameters. However, as an essential next step, these simulation results require experimental validation. Following successful validation, future research can explore the effects of different collimator designs, beam energies, and other dose modulation techniques on both the resulting dose profiles and the biological outcomes.

This simulation study demonstrates the SIRMIO beamline’s suitability for pMBRT research. The system can generate spatially fractionated dose profiles, achieving relevant PVDRs even without a collimator and significantly higher PVDRs with a collimator for finer fractionation. Interlacing beams also present a promising approach for achieving a homogeneous target dose while maintaining dose contrast in normal tissue, a concept that the SIRMIO beamline is well suited to investigate experimentally. The current SIRMIO system operates at a relatively low dose rate, mainly due to the limited beam current available from the accelerator at lower energies, for example, around 0.2 nA at 70 MeV beam energy at the Danish Centre for Particle Therapy (DCPT). While this dose rate is sufficient for accurate dosimetric assessments, it is near the lower threshold required for in vivo pMBRT studies. The achievable dose rate also depends on several factors, including energy-dependent transmission, the number of delivered spots, target volume, beamline setting, and the level of dose homogeneity required. Future work will include experimental validation to assess the system’s beam delivery accuracy and dose distribution, followed by in vivo mouse experiments. During these experiments, animals will be anesthetized to minimize movement, and irradiation will initially target relatively stable regions such as the mouse foot or brain to reduce the effect of organ movement. These planned dosimetric evaluations will help identify the system’s practical limitations and guide further optimization for pMBRT applications.

## Data Availability

Dose distributions produced with the MSC can be made available upon reasonable request to the first author at DCPT. Information on the SIRMIO platform and related data can be requested to the senior author from LMU.
